# QUALITY OF LIFE OF GRANDPARENTS RAISING GRANDCHILDREN IN NIGERIA

**DOI:** 10.1093/geroni/igz038.1065

**Published:** 2019-11-08

**Authors:** Joshua O Aransiola, Funmi Togonu-Bickersteth, Kolawole Aliyu, Mojirayo Afolabi, Akanni Akinyemi

**Affiliations:** 1 Obafemi Awolowo University, Ile-Ife, Ile-Ife, Osun State, Nigeria; 2 Obafemi Awolowo University, Ile-Ife, Osun State, Nigeria

## Abstract

This article examined the personal and household characteristics influencing the quality of life (QoL) of grandparents caring for grandchildren in Skipped Generation Households in Nigeria with a sample of 2, 144 grandparents in Imo, Lagos and Kano. Chi square and multinomial logistic regression were employed to understand the relationship between the dependent variable (QoL) and independent variables (personal and household characteristics). The level of the QoL of the grandparents almost spread evenly among low (34.3%), average (34.3%) and high (31.4%). Five domains of QoL were examined including level of independence (LI), psychological well-being (PW), social relation (SR), physical health (PH), environment (ENV) and engagement in income generating activities (IGA). Personal characteristics including; state of residence was significantly associated with all the domains except LI and PW, age was associated with IGA, LI and ENV, sex was associated with SR and ENV and level of education was associated with all the domains except IGA and ENV while religious affiliation was associated with IGA. Household characteristics including; sex of household head was significantly associated with PH, SR and ENV, age of household head was associated with IGA and LI and wealth index was associated with all the domains while the number of household members was significantly associated with ENV. The regression analysis shows that only state of residence and wealth index significantly influence the QoL of the grandparents (P≤0.05). The state of residence and wealth index are therefore important in any policy intervention for this category of elderly persons in Nigeria.

